# Successful Pregnancies after the Treatment of a Thymic Carcinoid

**DOI:** 10.1155/2015/802713

**Published:** 2015-07-29

**Authors:** Wiebren A. A. Tjalma

**Affiliations:** Department of Obstetrics and Gynecology, University Multidisciplinary Breast Clinic Antwerpen, Gynecological Oncology Unit, Belgium

## Abstract

The present report describes the case of a woman diagnosed with an adrenocorticotropic hormone- (ACTH-) secreting thymic carcinoid associated with Cushing's syndrome. Treatment consisted of tumour resection and 131-I-meta-iodobenzylguanidine (MIBG) therapy. In spite of her iatrogenic menopausal state she twice became pregnant and delivered two healthy babies but developed recurrences during both pregnancies. The last recurrence presented as a primary breast cancer. Despite poor prognosis our patient survived for eleven years. To our knowledge this is the first report of successful pregnancy and delivery in a patient with a thymic carcinoid.

## 1. Introduction

Thymic carcinoids are rare tumours and therefore the experience is limited [1–5]. They were differentiated from other thymic malignancies by Rosai and Higa [6]. Only 200 cases have been reported, with a strong male predisposition of at least 3 : 1 and an occurrence in all age groups, with a peak around 40 to 58 years [2, 5]. Thymic carcinoids have a malignant behaviour and are prone to local recurrences and lymph node as well as distant metastases [1, 3, 5]. The disease-free interval is generally short after initial surgery, usually limited to one or two years [7, 8]. Thymic carcinoids can also be associated with endocrinopathies, including the multiple endocrine neoplasia type I syndrome [3, 7]. The development of Cushing's syndrome due to ectopic adrenocorticotropic hormone (ACTH) secretion by a thymic carcinoid is exceedingly rare [9].

Our report will focus on the long-term evolution of a patient with an ACTH-secreting thymic carcinoid associated with Cushing's syndrome especially in relation to the obstetric outcome.

## 2. Case Report

The patient was diagnosed with Cushing's syndrome due to an ACTH-secreting carcinoid of the thymus at the age of 26. She presented with complaints of weight gain, general fatigue, muscle weakness, and amenorrhea. At physical examination a typical cushingoid appearance with moon face and hirsutism was apparent.

Free urinary cortisol was increased at 165 *μ*g/day (normal, 20–90 *μ*g/day), plasma ACTH was in the high-normal range at 48 ng/L (normal, 10–50 ng/L), and serum cortisol was not suppressed by 1 mg dexamethasone overnight (124 *μ*g/L; normal <3 *μ*g/L). An ectopic ACTH production was suspected since inferior petrosal sinus sampling did not demonstrate an ACTH gradient with the peripheral measurement. Scintigraphy with 111-In-octreotide (Octreoscan) revealed a mediastinal mass probably originating from the thymus and this finding was confirmed by CT scanning. The patient underwent a total thymectomy through median sternotomy. The surgical margins were microscopically invaded by tumoural cells. Pathological examination showed a thymic carcinoid with immunohistochemical positivity for ACTH. During a disease-free interval of approximately four years the free cortisol excretion remained normal and the cortisol secretion could be suppressed by dexamethasone. CT scanning and Octreoscan during this period did not show any abnormalities.

At the age of 30 she unexpectedly became pregnant. At 34 weeks of gestation she suffered from premature contractions and developed preeclampsia. She was referred with extreme hypokalemia and hyperglycemia and she developed an acute psychosis shortly after. Free cortisoluria was highly increased at 1020 *μ*g/day. A caesarean section was performed for maternal reasons and she gave birth to a healthy baby. All metabolic abnormalities resolved in the postpartum period and the free cortisoluria normalized. Nevertheless, CT scanning showed a mediastinal mass highly suspicious for a local recurrence. Resection of different lymph nodes was performed three months later by a posterolateral thoracotomy. Capsular breakthrough of the lymph nodes and invasion of an anonymous vein and the left mammary artery by a carcinoid were identified.

The following three years the patient remained free of symptoms, with normal ACTH and cortisol concentrations and without radiological signs of metastases, until she again rather acutely felt distressed. Free cortisoluria was increased to 200 *μ*g/day and plasma ACTH to 500 ng/L. Metastatic spread along the subclavian artery and retrosternally was confirmed by CT scanning and Octreoscan. As surgery was no longer feasible, the patient received four treatments with 150 mCi 131-iodine-meta-iodobenzylguanidine (131-I-MIBG) over a period of two years, resulting in a temporary improvement of the endocrine parameters although without normalization. As a result of this treatment, she developed hypothyroidism and went into menopause, for which she received thyroxine and estrogen-progestogen substitution. When administration of ketoconazole could no longer adequately control the cortisol production, the patient underwent a bilateral adrenalectomy and she received a substitutive therapy with hydrocortisone.

**Figure 1:**
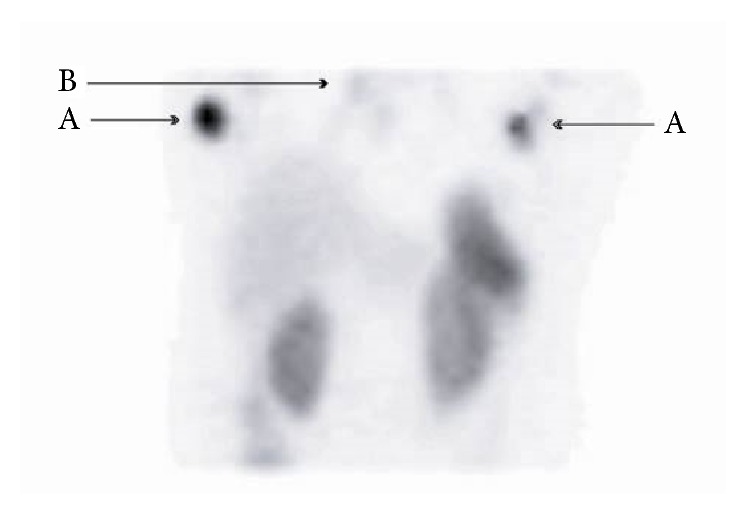
Whole body 111-In-octreotide scintigraphy showing uptake in both breasts (A), complementary to a mediastinal lesion (B).

Two months after the last treatment with 131-I-MIBG, the patient complained of abdominal distension. Ultrasonography showed an intact pregnancy with a gestational age about 18 weeks. Obstetric treatment consisted of careful follow-up and additional corticosteroid administration at 28 weeks of gestational age. She underwent a caesarean section because of foetal distress at the gestational age of 37 weeks and she delivered a healthy child. Four months after delivery the patient developed rapidly growing bilateral breast masses and enlargement of axillary lymph nodes. Mammograms and ultrasound examination showed bilateral multiple lesions; additional core biopsies suggested a ductal adenocarcinoma in both breasts. CT scan showed a mediastinal mass and lung metastases. Octreoscan revealed enhanced tracer uptake in the mediastinal lesion, in the thyroid gland, and in both breasts (Figure 1). Surgical excision was performed as bilateral modified mastectomy with axillary lymphadenectomy in combination with a thyroidectomy. Pathologic examination demonstrated multicentric localization of an atypical carcinoid in both breasts and thyroid with strong immunoreactivity for ACTH. The core biopsy specimens were revised and the initial diagnosis remained the same in spite of the definite diagnosis of a carcinoid tumour. At the age of 37 the patient developed a superior vena cava syndrome, confirmed by a CT scan showing compression of the trachea. Plasma ACTH levels were extremely elevated at 10700 ng/L. She was admitted for chemotherapy with etoposide, ifosfamide, and cisplatin, but she developed a neutropenic septic shock after one week. Due to recurrent infections the patient's situation deteriorated and she died one month later.

## 3. Discussion

There are some remarkable points of discussion in regard to this case of a thymic carcinoid. First we would like to point out that our patient is a relatively young woman. As mentioned, there is a strong male predisposition and the reported mean age is older [2, 3, 5]. A second issue is that our patient had a relatively long-term survival of eleven years, despite several local and distant metastases. Tiffet and colleagues described that the extent of the resection is a major prognostic factor [5]. In a series of 81 cases, the standard therapy of total excision is associated with 5-year and 10-year survival rates of 77% and 30%, respectively [7, 8]. However, other series report less prolonged survival rates between 10 and 28% [10].

Because of therapy with radioactive MIBG we were able to suppress the local recurrences for a number of years. The use of radiolabelled somatostatin analogues as therapy is described by Kaltsas and colleagues as a promising modality in the management of inoperable gastroenteropancreatic neuroendocrine tumours [11].

This type of therapy is associated with fewer side effects compared with systemic chemotherapy and the results have shown tumour stabilization and regression. The role of this therapy in the treatment of thymic carcinoids requires further investigation. Dosios and colleagues reported encouraging results in two patients with neuroendocrine thymic tumours treated by octreotide LAR [12].

The role of adjuvant chemotherapy has not been defined due to the low number of cases. On the contrary, chemotherapy has not been proven to be effective in eradicating the tumour nor in preventing local recurrences or distant metastasis. Therefore, the use of adjuvant chemotherapy should not be encouraged.

The unusual pattern of metastases of this carcinoid is the third point of discussion we want to focus on. Carcinoids are known for their frequent local recurrences and metastatic behaviour. Metastasis to lung and bone has been reported in as many as 70% of the cases, but the literature does not report on thymic tumours metastasizing to the breasts [3, 5]. Interestingly, thirteen cases of gastroenteropancreatic carcinoids metastatic to the breast have been described [13]. In contrast with the classical pattern of metastasis from the breasts to the axillary lymph nodes, this tumour metastasized inversely from mediastinal lymph nodes.

Our patient developed recurrences following both pregnancies after a disease-free interval of a couple of years. We can suggest that the progression of her disease is related to the elevated oestradiol or progesterone levels during the pregnancies. There are several reports on neuroendocrine tumours of breast but also of nonbreast origin with reactivity for oestrogen and progesterone receptors [14, 15].

We would also like to emphasize the fact that, despite the patient's history of cancer and her iatrogenic menopausal state after several MIBG treatments, she twice became pregnant. Her second pregnancy in particular was unexpected, since she had undergone an Octreoscan and MIBG therapy in the beginning of this pregnancy. The effect of this scintigraphy and MIBG treatment on the foetus is not known but in our case both babies were healthy without any foetal abnormality and they grew up normally. A literature study revealed that this is the first report of a successful pregnancy in a patient with an evolving thymic carcinoid.

## References

[1] de Montpreville V. T., Macchiarini P., Dulmet E. (1996). Thymic neuroendocrine carcinoma (carcinoid): a clinicopathologic study of fourteen cases. *Journal of Thoracic and Cardiovascular Surgery*.

[2] Filosso P. L., Dato G. M. A., Ruffini E., Bretti S., Dzzello F., Mancuso M. (2004). Multidisciplinary treatment of advanced thymic neuroendocrine carcinoma (carcinoid): report of a successful case and review of the literature. *Journal of Thoracic and Cardiovascular Surgery*.

[3] Fukai I., Masaoka A., Fujii Y. (1999). Thymic neuroendocrine tumor (thymic carcinoid): a clinicopathologic study in 15 patients. *Annals of Thoracic Surgery*.

[4] Talton C. C., Hopkins J. O., Walley B. D., Kincaid E. H. (2005). Metastatic thymic carcinoid: a case report. *The American Surgeon*.

[5] Tiffet O., Nicholson A. G., Ladas G., Sheppard M. N., Goldstraw P. (2003). A clinicopathologic study of 12 neuroendocrine tumors arising in the thymus. *Chest*.

[6] Rosai J., Higa E. (1972). Mediastinal endocrine neoplasm, of probable thymic origin, related to carcinoid tumor. Clinicopathologic study of 8 cases. *Cancer*.

[7] Detterbeck F. C., Parsons A. M. (2004). Thymic tumors. *Annals of Thoracic Surgery*.

[8] Gal A. A., Kornstein M. J., Cohen C., Duarte I. G., Miller J. I., Mansour K. A. (2001). Neuroendocrine tumors of the thymus: a clinicopathological and prognostic study. *Annals of Thoracic Surgery*.

[9] de Perrot M., Spiliopoulos A., Fischer S., Totsch M., Keshavjee S. (2002). Neuroendocrine carcinoma (carcinoid) of the thymus associated with Cushing's syndrome. *Annals of Thoracic Surgery*.

[10] Moran C. A., Suster S. (2000). Neuroendocrine carcinomas (carcinoid tumor) of the thymus: a clinicopathologic analysis of 80 cases. *American Journal of Clinical Pathology*.

[11] Kaltsas G. A., Papadogias D., Makras P., Grossman A. B. (2005). Treatment of advanced neuroendocrine tumours with radiolabelled somatostatin analogues. *Endocrine-Related Cancer*.

[12] Dosios T., Nikou G. C., Toubanakis C., Filippides T., Papachristou D. (2005). Multimodality treatment of neuroendocrine tumors of the thymus. *Thoracic and Cardiovascular Surgeon*.

[13] Rubio I. T., Korourian S., Brown H., Cowan C., Klimberg V. S. (1998). Carcinoid tumor metastatic to the breast. *Archives of Surgery*.

[14] Jochems L., Tjalma W. A. A. (2004). Primary small cell neuroendocrine tumour of the breast. *European Journal of Obstetrics Gynecology and Reproductive Biology*.

[15] Keshgegian A. A., Wheeler J. E. (1980). Estrogen receptor protein in malignant carcinoid tumor. A report of 2 cases. *Cancer*.

